# Case report: Anterior mediastinal mass in a patient with pleural effusion and dyspnea

**DOI:** 10.3389/fsurg.2023.1088642

**Published:** 2023-05-03

**Authors:** Lin Gu, Gaojie Xin, Yue Qu, Kai Wang, Ke Jiang, Shijie Xing, Ai Huang

**Affiliations:** ^1^ Department of Thoracic Surgery, Huazhong University of Science and Technology Union Shenzhen Hospital, Guangdong, China; ^2^ Department of Thoracic Surgery, Union Hospital, Tongji Medical College, Huazhong University of Science and Technology, Wuhan, China

**Keywords:** anterior mediastinal mass, composite lymphoma, dyspnea, gene rearrangement, complete response

## Abstract

**Background:**

Compound lymphoma is an uncommon type of lymphoid malignancy, and those consisting of concurrent B- and T-cell tumors are relatively rare.

**Case Summary:**

A 41-year-old man was presented with a 1-month history of progressively worsening cough, chest tightness, and dyspnea after exercise, which could be relieved following rest. Contrast-enhanced computed tomography scan demonstrated a 7.4 × 4.9 cm^2^ heterogeneous mass in the anterior mediastinum, where a large area of cystic liquid existed, and multiple enlarged lymph nodes in the mediastinum. Since the biopsy failed to yield an exact diagnosis and there was no sign of metastasis, the tumor was surgically resectioned. Surgical findings included obscure boundaries and consistent tumor stiffness with pericardial and pleural invasion. Further pathological examination combined with immunophenotype and gene rearrangement test found the mass composite of angioimmunoblastic T-cell lymphoma (AITL) and B-cell lymphoma. The patient recovered well after R0 resection and received chemotherapy with four cycles of CHOP combined with chidamide 2 weeks after surgery. The patient has had a complete response for over 60 months.

**Conclusion:**

In conclusion, we reported a composite lymphoma of AITL combined with B-cell lymphomas. Our experience provides the first successful attempt to treat this rare disease with combined surgery and chemotherapy.

## Case presentation

### History of illness and physical examination

A 41-year-old man was presented with a 1-month history of progressively worsening cough, chest tightness, and dyspnea after exercise, which could be relieved following rest. He denied any history of smoking, radiation exposure, known lung disease, or a personal or family history of malignancy.

The patient visited the Department of Respiratory Medicine for treatment due to the rapid development of dyspnea and worsening cough. Upon admission, he presented a dry cough and slight fever without chest pain, hemoptysis, palpitations, nausea, vomiting, abdominal pain, diarrhea, or other symptoms. Physical examination revealed unpalpable lymph nodes in the supraclavicular fossa bilaterally. Laboratory examinations were normal, including complete blood count, liver and kidney function, and electrolyte levels. The abdominal ultrasonography did not reveal any abnormality.

### Imaging examination

**Figure 1:**
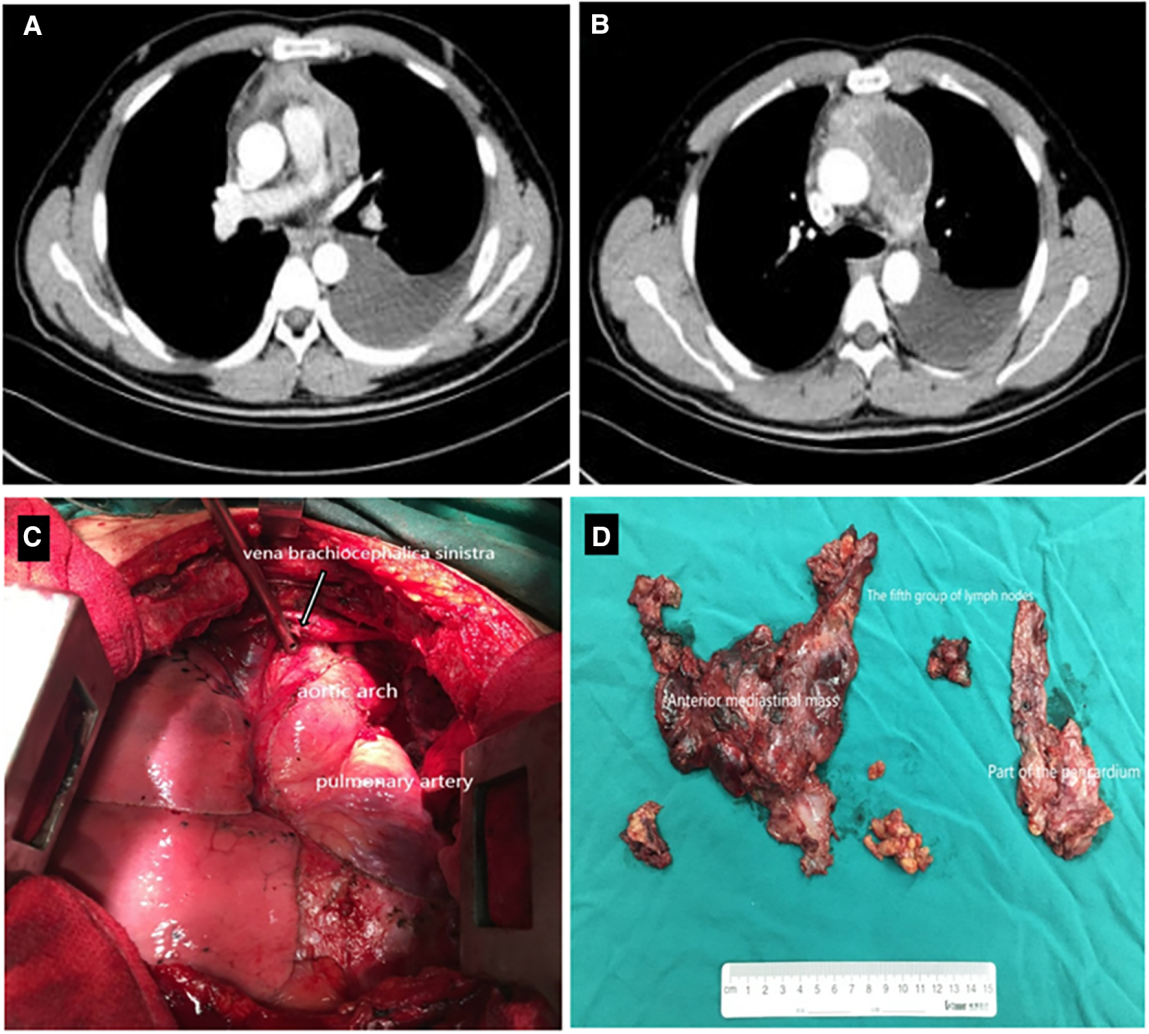
Contrast-enhanced CT scan demonstrated a 7.4 × 4.9 cm^2^ heterogeneous mass in the anterior mediastinum, where a large area cystic liquid existed, and multiple enlarged lymph nodes in the mediastinum (**A,B**); The whole mass, invaded tissues and adjacent enlarged lymph nodes were removed completely (**C,D**). CT, computed tomography.

**Figure 2:**
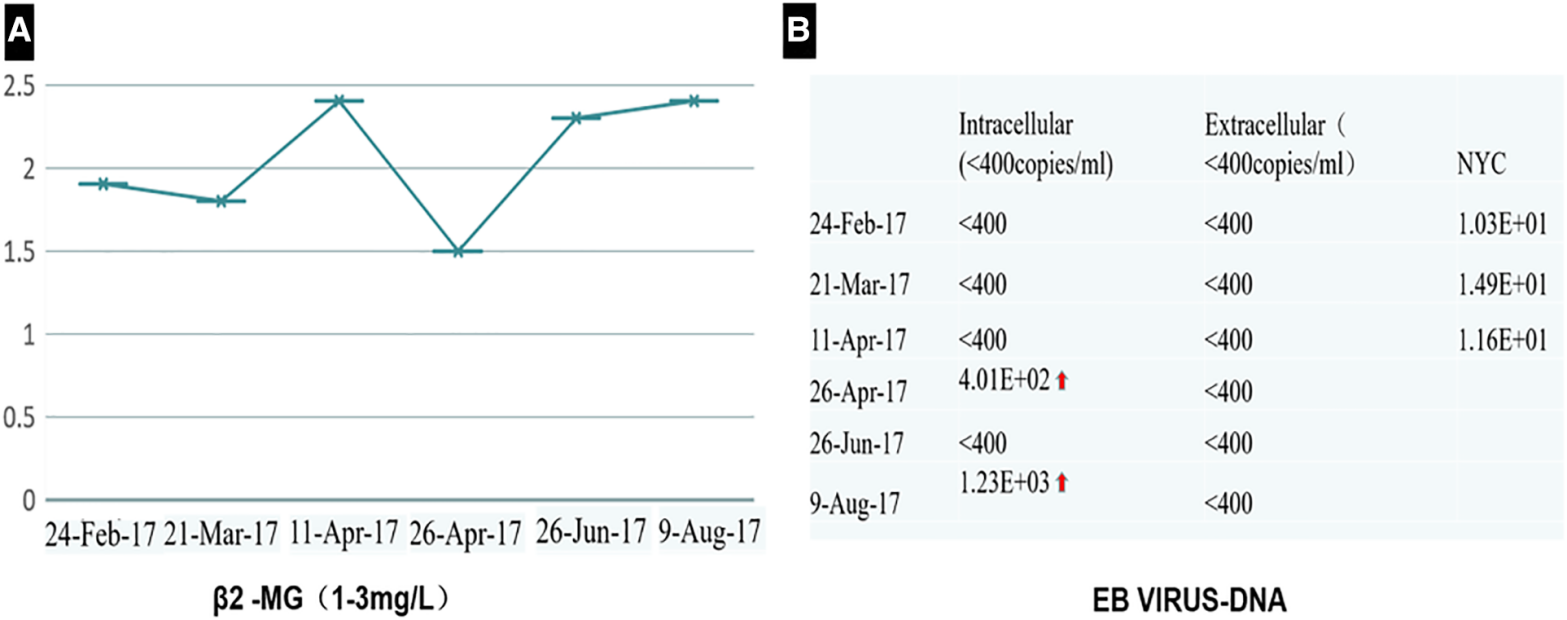
The test of *β*2 MG (**A**) and EBV-DNA (**B**) showed that they were less than the standard limit in half a year follow-up.

Contrast-enhanced computed tomography (CT) scan demonstrated a 7.4 cm × 4.9 cm heterogeneous mass in the anterior mediastinum, where a large area of cystic liquid existed, and multiple enlarged lymph nodes in the mediastinum (Figure 1). The mass was considered to be a malignant tumor. Pleural effusion was drained and collected for an exfoliative cytology test, but no tumor cells were found. Meanwhile, EBV-DNA was less than 400 copies/ml (reference range <400 copies/ml), and β2 MG was 1.9 mg/l (reference range 1.0–3.0 mg/l) (Figure 2). Needle biopsy showed fibrous tissue and hyperplasia of granulation tissue with chronic inflammatory cell infiltration, including neutrophils and eosinophils.

### Treatment and final diagnosis

**Figure 3:**
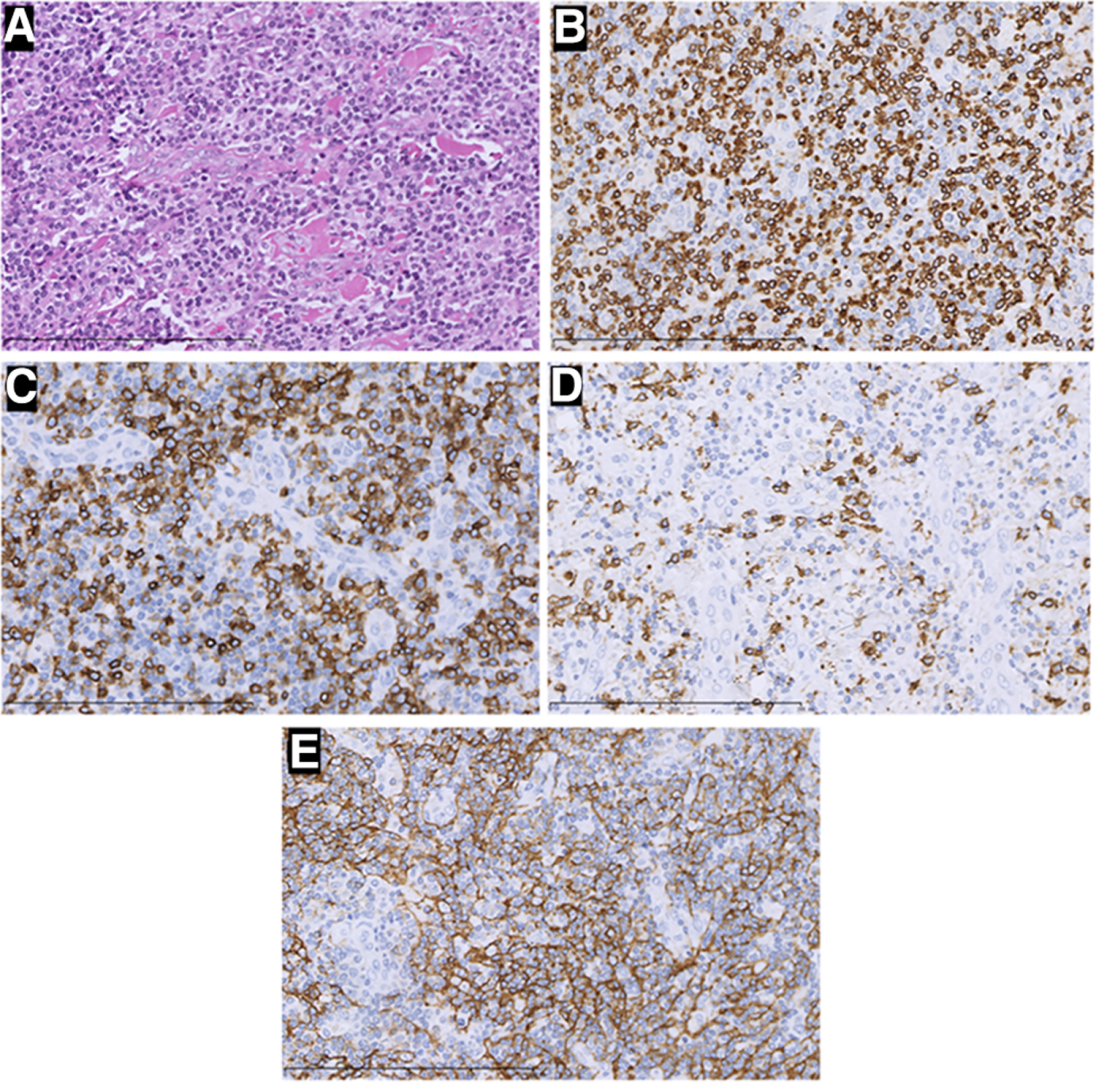
H&E staining and immunohistochemistry pictures of the mass (×400). In this part, H&E staining picture revealed a diffuse infiltration of tumor cells that were rich in plasma and possessed vesicular chromatin accompanied prominent nucleoli (**A**). The tumor tissue strongly expressed CD3 (**B**), PD-1 (**C**), and CD21(**E**), but was negative for CD20 (**D**). Those were considered angioimmunoblastic T-cell lymphomas.

**Figure 4:**
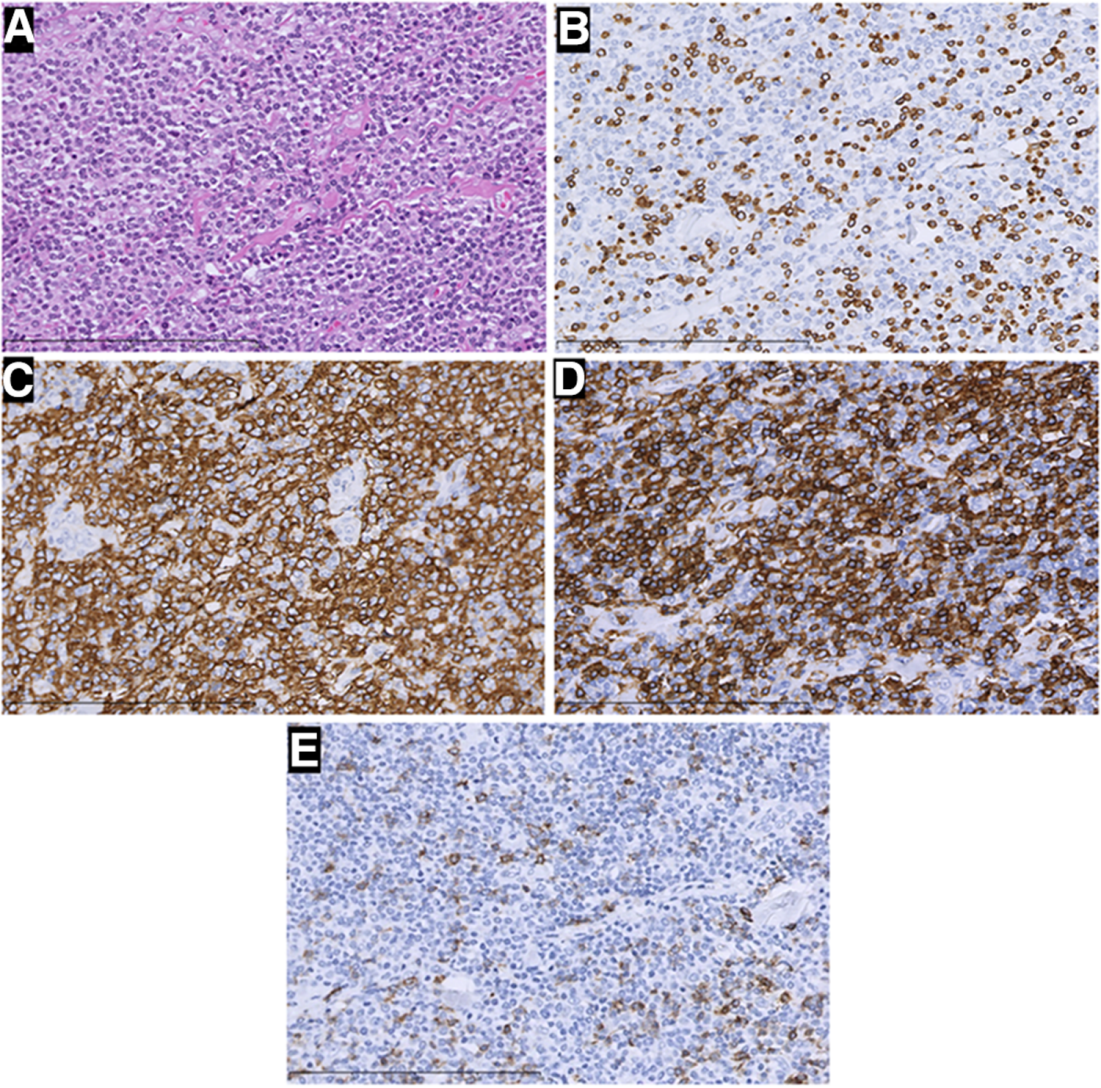
H&E staining and immunohistochemistry pictures of the mass (×400). In this part, H&E staining picture revealed a diffuse infiltration of tumor cells that were rich in plasma, and possessed vesicular chromatin accompanied prominent nucleoli (**A**). The tumor tissue strongly expressed CD20 (**C**) and CD43 (**D**), but was negative for CD3 (**B**) and PD-1 (**E**) Those were considered B-cell lymphomas.

**Figure 5:**
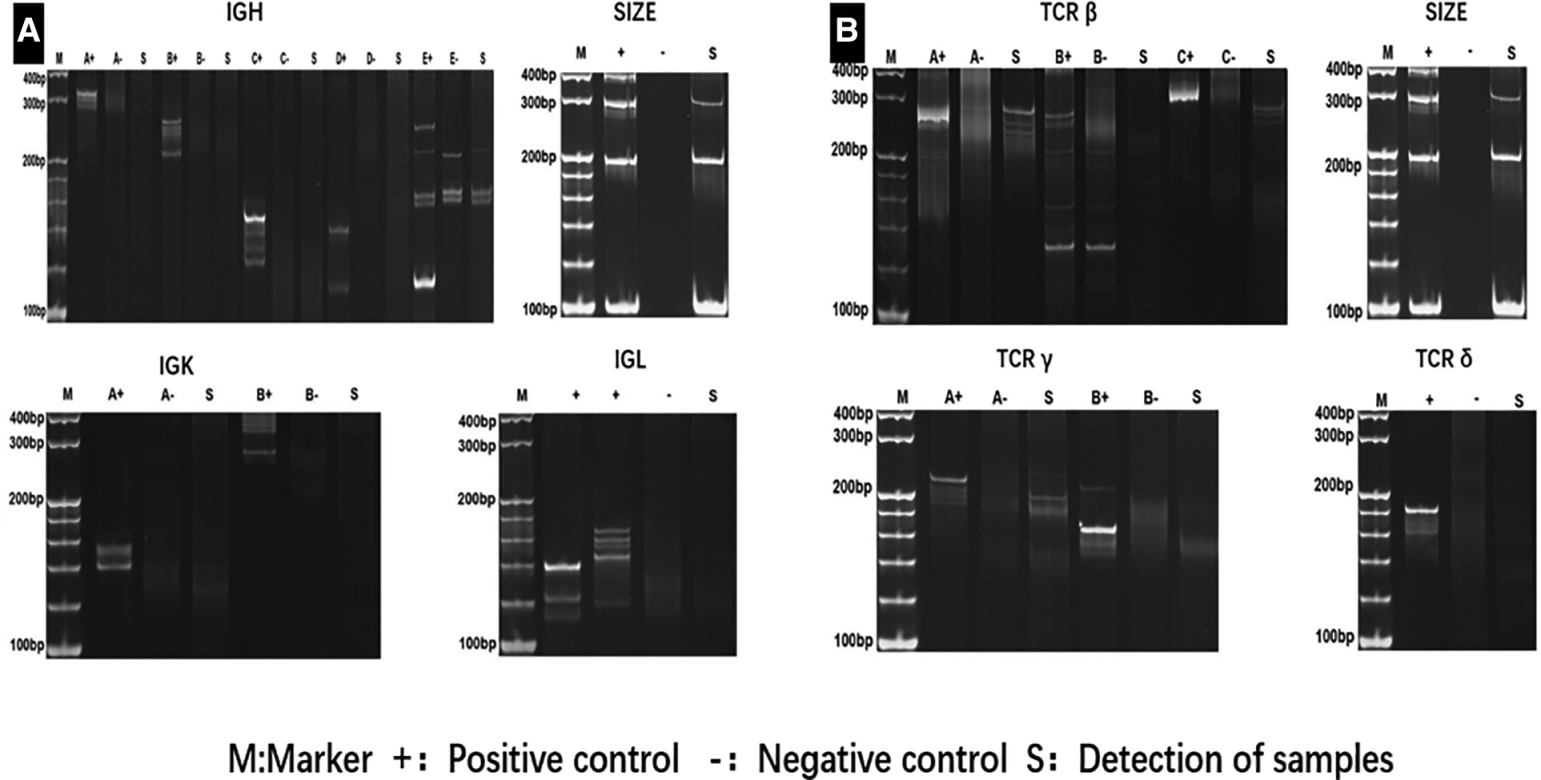
Gene rearrangement test revealednot *IGH*, *IGK*, and *IGL* (**A**), but *TCRγ*, *TCRδ* and *TCR*β (**B**) cloning rearrangement.

Two times, Flexirigid thoracoscopy biopsies did not obtain the tumor parenchyma due to the inflammation and hyperplasia around the mass. Then, the patient was transferred to the Department of Thoracic Surgery. Middle thoracotomy was performed under general anesthesia. Surgical findings included obscure boundaries and consistent tumor stiffness with pericardial and pleural invasion. The whole mass invaded tissues, and adjacent enlarged lymph nodes were eradicated (Figure 1). Intraoperative consultation showed R0 resection. Postoperative histology showed nodules separated by collagen fibers in the tumor. There were two morphologically and immunophenotypically distinct components–T- and B-cell mixed hyperplasia (Figure 3 and 4). Further pathological examination combined with immunophenotype and gene rearrangement test found the mass composite of angioimmunoblastic T-cell lymphoma (AITL) and B-cell lymphoma. Immunophenotypic analyses showed the lymphocytes were CD5+, CD3+, CD10 (partial +), PD1+, CD43+, BcL2+, CD21 and CD23 (FDC net irregular hyperplasia), MUM1−, CD30+ (scattered in large cells), TdT−, CD56−, PCK+ (thymic epithelium), CD15−, CyclinD1−, Bcl6−, IgD−, Kappa−, Lambda−, CD20+ and PAX5+ (B cells), and CD43+ (diffuse, B cells with abnormal expression) (Figure 3 and 4). Gene rearrangement test revealed *TCRG* and *TCRB*, but not *IGH*, *IGK*, or *IGL*, cloning rearrangement (Figure 5).

### Outcomes and follow-up

**Figure 6:**
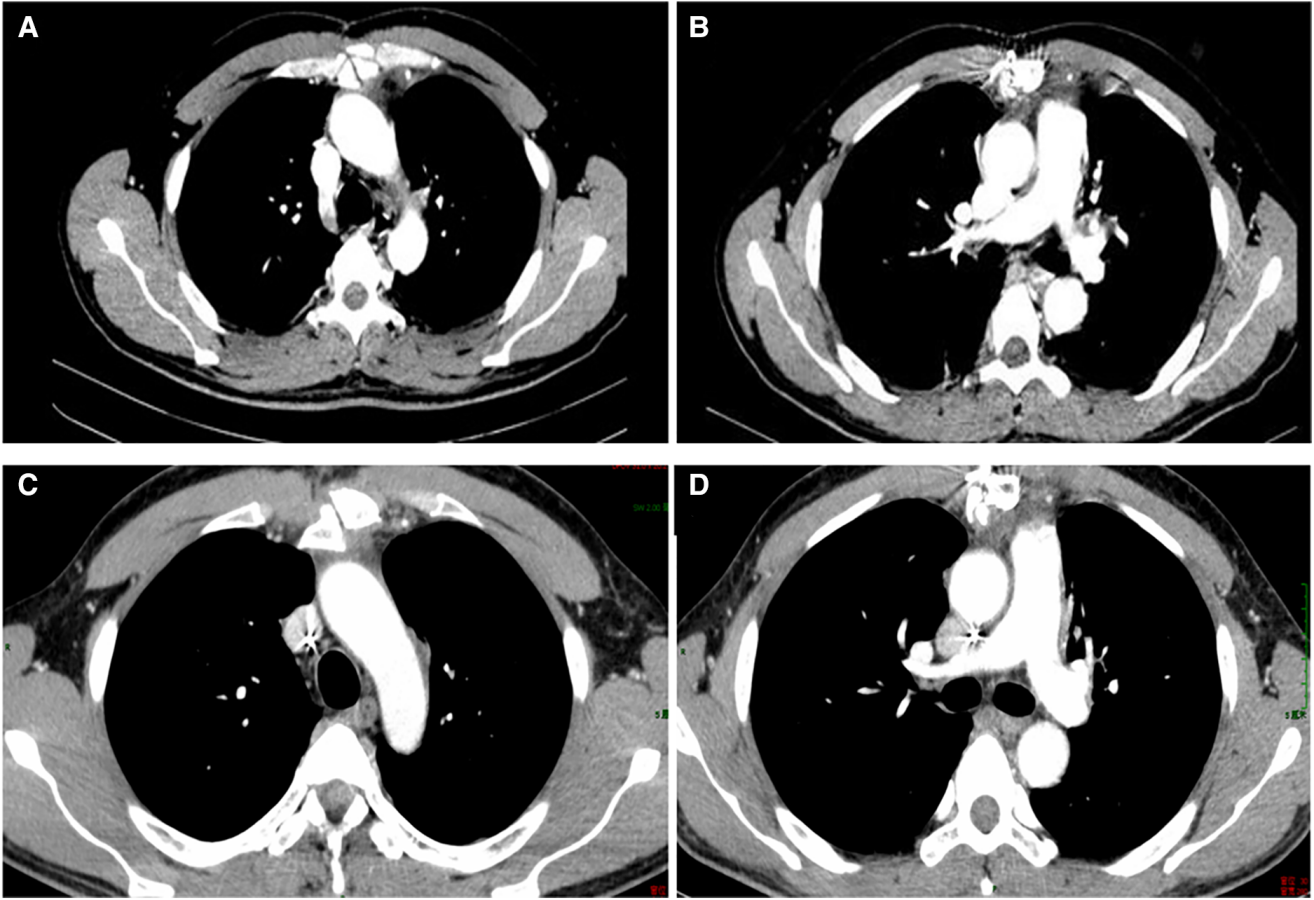
CT in the 1-month follow-up (**A,B**); CT in the 7-month follow-up (**C,D**). CT, computed tomography.

The patient recovered well after R0 resection and received chemotherapy with four cycles of CHOP combined with Chidamide 2 weeks after surgery. The test of EBV-DNA, β2 MG (Figure 2), and CT (Figure 6) showed they were less than the standard limit in half a year follow-up. The patient has had a complete response (CR) for over 60 months.

## Discussion

Compound lymphoma (CL) is an uncommon type of lymphoid malignancy, and those consisting of concurrent B- and T-cell tumors are relatively rare. The definition of CL was first proposed by Custer (1) and later improved by Kim et al. (2). CL is defined as two or more morphologically and immunophenotypically distinct lymphomas or lymphoid neoplasms that occur in the same organ or tissue (3). About 1%–4% of lymphomas are CL (4). Various types of CL have been described in the literature, including the combinations of all the major types of lymphomas, while most reported cases were B-cell lymphomas combined with Hodgkin lymphomas or two composite B-cell lymphomas of different types (5–7).

Cutting-edge research suggests that diverse mechanisms may underlie the coexistence of two lineages in a single patient. For example, a possible reason is a bidirectional differentiation after malignant transformation at the hematopoietic stem or progenitor cell level (8, 9). It has been well documented since the early 1980s that these pluripotent cells with the capacity to differentiate into a B- or T-cell lineage underwent *TCR* and *IGH* gene rearrangements. T-cell gene rearrangements occur not infrequently in B-cell lymphomas (10–15), while immunoglobulin heavy chain gene rearrangements occur in T-cell neoplasms (12, 16, 17). Chronic viral infections and immunodeficiencies may be two causative factors (18, 19). The specific microenvironment in AITL might allow the unrestricted expansion of EBV-infected B cells, increasing the risk of developing an EBV-positive B-cell lymphoma. However, no single pathogenic mechanism likely applies to all cases. Our patient did not have a predisposing immunological condition. Gene rearrangement of *TCRB* and *TCRG*, but not *IGH*, *IGK*, or *IGL*, indicated the emergence of separate clones from separate cells of origin. Furthermore, both components in the tumor were negative for EBV, which eliminated the role of this virus in causing the disease.

Due to the complexity of dealing with multiple types of lymphoma simultaneously, therapeutic goals for CL vary. Despite increasingly intense treatments, the outcome of patients with T-cell non-Hodgkin lymphoma remains unsatisfactory (20, 21). The overall therapeutic strategy needs to consider both disease components in CL. Given the rarity and heterogeneity of CL, reliable data for the natural history and appropriate treatment are unavailable, with published work mainly focusing on reporting biological features of individual cases. Nevertheless, available data suggest that two or more components of CL behave similarly to the respective entities alone. These components will also determine the therapeutic strategy (2, 22, 23). Irrespective of histological types, most present first-line chemotherapy protocols are based on alkylating agents. Patient survival is enormously affected by the response of the more aggressive T-cell component to chemotherapies, including CHOP [cyclophosphamide, doxorubicin (hydroxy daunomycin), vincristine (Oncovin), and prednisone], CHOP-like regimens ± rituximab, and fludarabine ± alemtuzumab (7, 24).

In our case, the patient had been previously healthy until the onset of dyspnea and cough. CT identified a large heterogeneous mass in the anterior mediastinum with massive pleural effusion. However, cytopathology failed to reveal tumor cells, and pleural effusion was reactive. The patient was preliminarily diagnosed with a thymic tumor or lymphoma. Given that the biopsy did not reach a precise diagnosis, we took a surgical procedure to establish the diagnosis and ease symptoms. Finally, we diagnosed the mass as non-Hodgkin's angioimmunoblastic T-cell lymphoma with B-cell lymphoma according to pathological and immunophenotyping results and DNA clonal analysis of gene rearrangements.

Most people deem that surgery is not the primary therapeutic strategy for CL. However, surgery is warranted in this case because of the symptoms of massive pleural effusion, the lack of apparent systemic lymphadenopathy or enlarged liver and spleen, and the failure of a biopsy to reach a reliable diagnosis.

During a 3-month (ranging from 1–13 months) follow-up of CLs patients with AITL and diffuse large B-cell lymphoma (DLBCL) who received chemotherapy, 52% of patients died (7). Due to the complexity of treating multiple types of lymphoma simultaneously, CL presents challenges with treatment and assessing prognosis. Our cases show that surgery is an effective way to diagnose and treat difficult-to-diagnose lymphomas in countries or regions where the precise techniques and equipments are lacking.

## Conclusions

In conclusion, we reported a composite lymphoma of AITL combined with B-cell lymphomas. Our experience provides the first successful attempt to treat this rare disease with combined surgery and chemotherapy.

## Data Availability

The datasets presented in this study can be found in online repositories. The names of the repository/repositories and accession number(s) can be found in the article/supplementary material.
